# Supplementary material for: The EuropaBON Stakeholder Dashboard: A dynamic web application to map Europe’s biodiversity community
Source: PLoS One. 2025 Aug 13;20(8):e0329390. doi: 10.1371/journal.pone.0329390 (PMC12349692; doi:10.1371/journal.pone.0329390)
Supplement: S2 File — (DOCX) [file pone.0329390.s002.docx]

The entries for the field "Indicate your position in the biodiversity data flow" have been removed:

- We are in planning phase

- Various

- German federal states

- Multiple sources

- National databases

- Science and remote sensing data

- tba

- To be added soon

- Research purpose

- …

- The data we collecte in our monitoring scheme

- N/A

- My own

- n.a.

- Not clear yet, we will use hybrid data

- Many institutions

- many

- data from research institutions and other management authorities

- Many governments and businesses

- Universities

- ICP Forests member states

- BLI partnership

- EU pollinator monitoring

- NGOs

- several

- Overall end-users

- Local government

- data not published yet, still in validation phase

- not providing at the moment, developing tech

- No specific institution

- on request

- Various entities

- Any user

- Stakeholders, farmers

- industry, academia, citizen science, policy, open access

- variouse dataportals, datapapers

- ,

- ;

- Independent researcher

- Myself. I collected data for my Ph.D. which I am using.

The entries for the field "Indicate your position in the biodiversity data flow" have been corrected:

- value by the user -> corrected value by the authors

- Remote Sensing (Copernicus etc.) -> Copernicus

- GBIF -> Global Biodiversity Information Facility (GBIF)

- EEA -> European Environment Agency (EEA)

- Alma Mater Studiorum University of Bologna -> University of Bologna

- LUCAS dataset -> LUCAS

- European Vegetation Archyve -> European Vegetation Archive (EVA)

- Natuurpunt.Studie -> Natuurpunt Studie

- waarnemingen.be -> natuurpunt studie

- Copernicus Global Land Service -> Copernicus

- WFD data -> WISE Water Framework Directive Database

- Executive Environment Agency -> Executive Environment Agency (ExEA)

- CSIC -> The Spanish National Research Council (CSIC)

- Edaphobase -> Edaphobase Query Portal

- Stazione Zoological A. Dorhn -> Stazione Zoologica Anton Dohrn

- Centre de Ciència i Tecnologia Forestal de Catalunya -> Forest Science and Technology Centre of Catalonia (CTFC)

- Ciências - Universidade de Lisboa -> Faculty of Sciences of the University of Lisbon

- ISA University of Lisboa -> School of Agriculture University of Lisbon (ISA)

- MARE - Marine and Environmental Sciences Centre -> Marine and Environmental Sciences Centre (MARE)

- CONICET (UNJU)) -> UNJu - CONICET

- glonaf.org -> GloNAF (Global Naturalized Alien Flora)

- Pladias.cz -> Pladias (Czech Flora and Vegetation)

- EOMODNET and EUROBIS is planned -> EOMODNET,EUROBIS

- Online databank, e.g. NCBI -> NCBI

- Bundesamt für Naturschutz (German Federal Agency for Nature Conservation) -> Bundesamt für Naturschutz (BfN)

- Enviromental department affairs -> Department of Environmental Affairs, South Africa

- Public Service Wallonia -> Service Public de Wallonie (DEMNA)

- Ministry of Environment without country -> add correct name and country

- Institute of Landscape Ecology - SAS -> Institute of Landscape Ecology of SAS

- Milennium Biodiversity Institute of Antarctic and Sub-Antarctic Ecosystems (BASE) -> Millennium Biodiversity Institute of Antarctic and Sub-Antarctic Ecosystems (BASE)

- SWaM coordinates most of the marine & freshwater dataflow in Sweden, with multiple data pathways. -> Swedish Agency for Marine and Water Management (SWAM)

- SWaM is charged with the overall coordination of the marine & freshwater monitoring in Sweden, which depends on a multitude of annual financing agreements and dataflows, many of which SWaM also uses for internal, national and international purposes -> Swedish Agency for Marine and Water Management (SWAM)

VLIZ -> Flanders Marine Institute (VLIZ)
