# Supplementary material for: The EuropaBON Stakeholder Dashboard: A dynamic web application to map Europe’s biodiversity community
Source: PLoS One. 2025 Aug 13;20(8):e0329390. doi: 10.1371/journal.pone.0329390 (PMC12349692; doi:10.1371/journal.pone.0329390)
Supplement: S4 File — (DOCX) [file pone.0329390.s004.docx]

We have provided four API endpoints:

1. **Total statistics 
   Request:** <https://europabon.org/dashboard/api/total>  
   **Response:** Returns the total number of registered members, institutes, and countries.
2. **Projects and categories
   Request:** <https://europabon.org/dashboard/api/projects>  
   **Response:** Provides a list of key EU projects and infrastructures, categorised as follows: research infrastructures/networks/projects; coordination and support networks; biodiversity tools and technologies; data repositories; biodiversity observation frameworks/networks; biodiversity monitoring schemes; and intergovernmental organisations and panels.
3. **Regional classification
   Request:** [https://europabon.org/dashboard/api/regions?q={EU_region}](https://europabon.org/dashboard/api/regions?q=%7bEU_region%7d)  
   **Response:** Returns a list of countries within the specified EU region. For example, <https://europabon.org/dashboard/api/regions?q=western%20europe> returns the countries in Western Europe. This endpoint is used to show the network graph by EU region.
4. **Network graph**

**Request:** <https://europabon.org/dashboard/api/nodes>

**Response:** Provides a list of all institutes as nodes and their connections as edges. The information for the activity level is returned as follows: "events": {"total": "0-17", "level": "0-2"}. The information for the data position is returned as follows: "group": "data user/ data provider/ data user_provider/ N.A.". The edges in the network graph represent data exchange interactions between registered institutions. Edges between nodes use the labels “from” (data user) and “to” (data provider) and refer to the node IDs. While the network graph of the dashboard itself is displayed as an undirected graph, i.e. we do not show “from” and “to” directions in the network graph, e.g. as arrows. However, the pop-up window of each node in the network graph shows the information from which institution data is used or to which institution data is provided.
