# Supplementary material for: The EuropaBON Stakeholder Dashboard: A dynamic web application to map Europe’s biodiversity community
Source: PLoS One. 2025 Aug 13;20(8):e0329390. doi: 10.1371/journal.pone.0329390 (PMC12349692; doi:10.1371/journal.pone.0329390)
Supplement: S7 File — (DOCX) [file pone.0329390.s007.docx]

**Website:** <https://europabon.org/dashboard>

**Git Repository:** <https://github.com/EuropaBON/stakeholder-dashboard>

**Zenodo:** <https://doi.org/10.5281/zenodo.10047342>

**Usage license:** GNU General Public License Version 3 (GPLv3)

**Platform:** Cross-Platform

**Programming language:** HTML, JavaScript, CSS

**Interface language:** English
