# Supplementary figures and images for: The EuropaBON Stakeholder Dashboard: A dynamic web application to map Europe’s biodiversity community
Source: PLoS One. 2025 Aug 13;20(8):e0329390. doi: 10.1371/journal.pone.0329390 (PMC12349692; doi:10.1371/journal.pone.0329390)

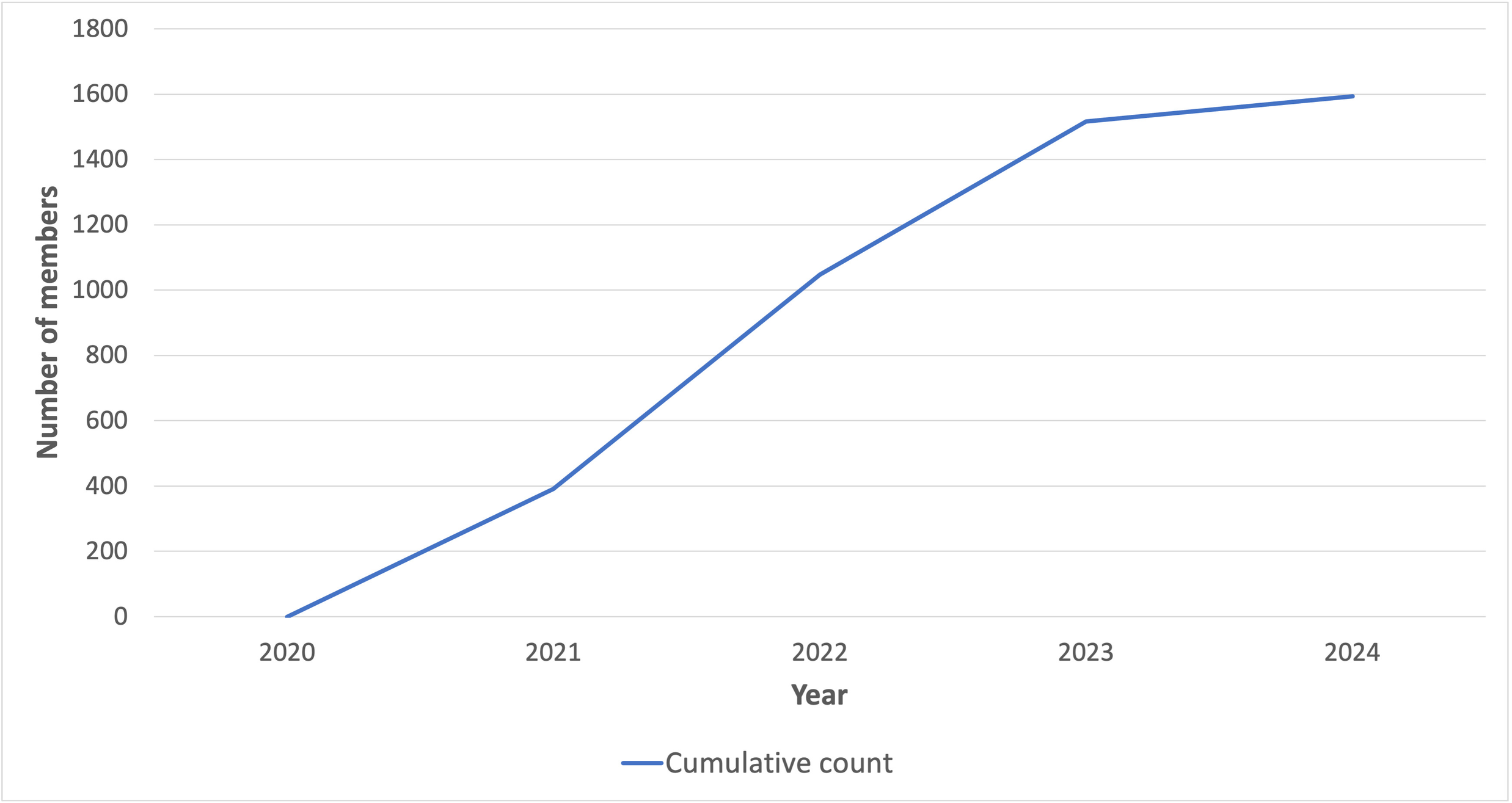

Supplement: S1 Fig — (TIF) [file pone.0329390.s008.tif]

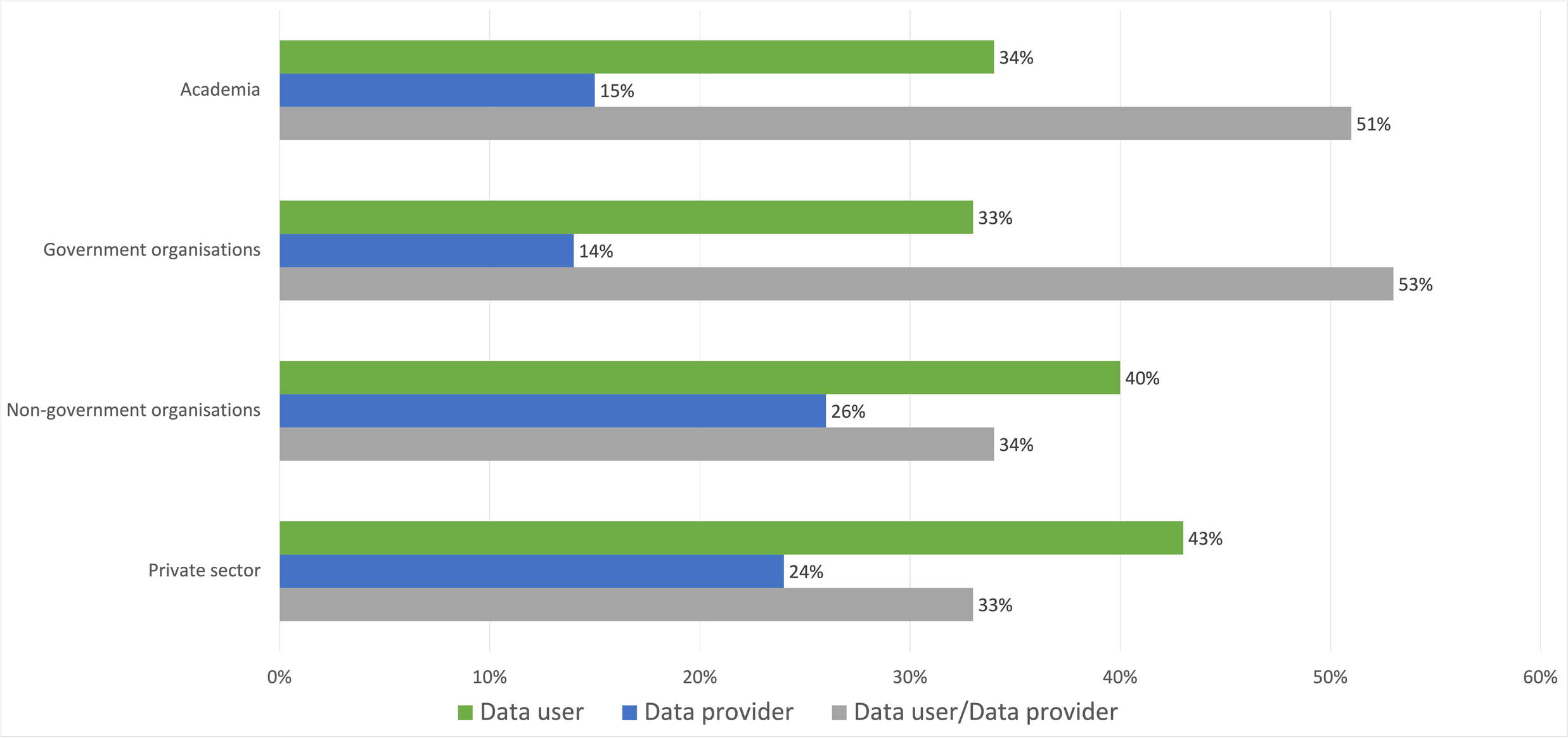

Supplement: S2 Fig — Citizen scientists are excluded from this figure due to the small number of stakeholders that belong to this occupational category. Stakeholders that could not be categorised into either of these groups were classified as “other” and are also excluded from this figure. (TIF) [file pone.0329390.s009.tif]

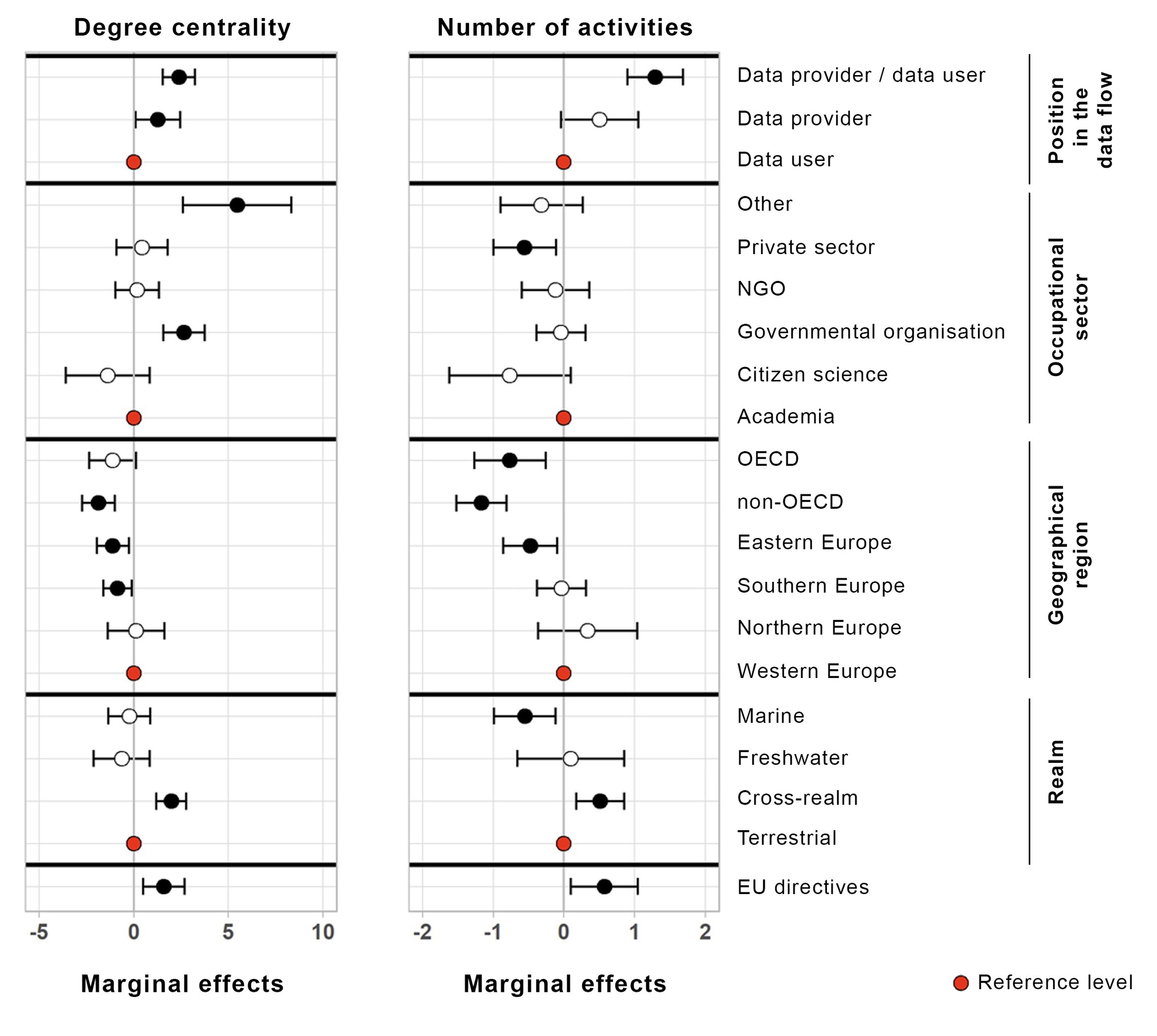

Supplement: S3 Fig — Marginal effects and corresponding 95% confidence intervals calculated for explanatory variables grouped into categories (i.e., position in the data flow, occupational sector, geographical region, realm, and EU directives). Dependent variables are stakeholder connectedness (degree centrality) and participation in EuropaBON stakeholder events (number of activities). Filled- and open circles indicate significant (p-value 0.05) and non-significant effects on degree centrality and number of activities that stakeholders participated in. Filled red circles indicate reference levels. (TIF) [file pone.0329390.s010.tif]
