# Supplementary material for: The EuropaBON Stakeholder Dashboard: A dynamic web application to map Europe’s biodiversity community
Source: PLoS One. 2025 Aug 13;20(8):e0329390. doi: 10.1371/journal.pone.0329390 (PMC12349692; doi:10.1371/journal.pone.0329390)
Supplement: Attachment — Submitted filename: Response to Reviewers.docx [file pone.0329390.s012.docx]

Response to Reviewers

*********************************************************************

**Reviewer #1:**

*********************************************************************

The manuscript "The EuropaBON Stakeholder Dashboard: A dynamic web application to map Europe's biodiversity community" provides excellent insight into the EuropaBON stakeholder community, which is essential for building a solid and comprehensive biodiversity monitoring network. The paper focusses on the analysis of stakeholder/member data (entered upon registration) and its visualisation through an interactive, and publicly accessible, online dashboard.

I believe this paper merits publication, but I feel it lacks specific details and clarity in writing when it comes to technical soundness and use of statistics.

One of the main questions pertains to the provenance of the data, which I consider essential for evaluating the representativeness of the results. This consists of two components:

* How have you collected information on the 'edges', i.e. which node contributes data to whom, and receives data from whom?

* How have you dealt with 'translating' information provided by individual members to institution/node level?

RESPONSE: Thank you for your comment. We've added a new sub-heading, "Data Processing," with detailed information to the API endpoints to better explain the creation of nodes and edges. Please see below for more information.

The connections in the network graph (i.e., the edges) represent data exchange interactions between registered institutions, categorized as biodiversity data users, providers, or both. This information was supplied by EuropaBON members during the registration process (<https://europabon.org/members/register/index>). A complete list of registration form questions is available in Appendix S2.

In the API response (<https://europabon.org/dashboard/api/nodes>), the edges are defined using the labels ‘from’ (data user) and ‘to’ (data provider), which refer to the respective node IDs. While the network graph is displayed as an undirected graph - meaning we do not visually indicate directionality with arrows - the pop-up window provides details on each node’s position within the data flow.

We have grouped the members into a table according to their institution (S4 file), i.e. several individual members can belong to the same institution and are therefore displayed as one and the same institution (i.e., node).

Some information about the node was provided by the members during the registration process (e.g., country, realm, etc.); other information (e.g., centrality and pageRank) was calculated using the software cytoscape.js based on each node's edges. The information of each node can be seen either in the table in the dashboard or as a pop-up window of each node in the network graph.

Also, please detail any actions you may have taken to assess the representativeness of this edges.

RESPONSE: We have not specifically taken any measures to assess representativeness of the edges (i.e., the information flow) and rely entirely on the information provided by the members during the registration process. As with any other free input field, errors such as incorrect or incomplete entries cannot be completely ruled out. We have included an Appendix S3 that shows examples of data cleansing measures for the data flow position entries (i.e. the edges in the network graph). However, no further assessment of the user entries has been carried out, so it is possible that the information is incomplete.

With regards to the statistical analysis, I am not familiar with the negative binomial model used, but -as detailed in the specific comments- miss a clear explanation of the methods and parameters used in this section.

RESPONSE: The entire section “Statistical analysis of network data” has been rewritten. We hope it is much clearer now.

Finally, I presume there’s more to learn from the available data using alternative / additional visualisations. It is no surprise that big (networked) organisations such as GBIF, data ‘requesters such as the EEA and EC and research infrastructures have a high level of centrality. Additionally for some of these nested/networked organisations both the overarching, as well as the regional node organisation feature among the nodes (e.g. OBIS - EurOBIS, [INSDC] - NCBI-Genbank / ENA). It is unclear how this is accounted for. Given the focus on biodiversity monitoring, I wonder if an analysis excluding/hiding those initiatives and having more focus on the institutes who perform actual ‘on the ground’ monitoring would provide additional insights into the European landscape. In it’s current form the visualisation for (1) occupational sectors and (2) realms have only limited information value, and (3) the geographic regions visualisation could possibly be improved by using the colour coding for variables other than geographic region (which is evident from the map).

RESPONSE:

Thank you for this comment. It is true that several large, overarching institutions have smaller, nested nodes within them. Whether a smaller organisation, such as a regional node, appears as an individual node in the network or is nested within a broader institution depends on the information provided by members during the registration process.

As outlined in the additional text on data processing, we made efforts to standardise the data as much as possible. However, we did not consistently list sub-organisations as individual nodes, as in many cases, there were no members representing these in our network. We acknowledge that this may limit the value of the visualisation tool to some extent. However, excluding or hiding overarching institutions, adding individual nodes for all sub-institutions, and systematically identifying institutions solely focused on biodiversity monitoring would exceed the intended scope of this tool.

Regarding the colors in the visualisation tool, we acknowledge that the color-coding of nodes representing the EU region may appear somewhat redundant when the network is displayed by the EU region. However, modifying the legend and color scheme is not a straightforward adjustment, as it would require a complete rebuild of the visualisation tool. Given these technical constraints, we have opted to maintain the current color scheme in the map.

**Specific comments**

* The main objectives include identifying skill gaps. I am unsure how this is covered by the data and analysis provided.

Thank you for this comment. We agree that the term “identifying skill gaps” may not reflect the study’s focus accurately. The primary objectives of our work were to analyse stakeholder engagement and influence within the network, and to identify potential geographical, thematic, and sectoral gaps. An additional goal was to present this information in an accessible format through an interactive, dynamic dashboard that visualises the stakeholder network and enables real-time exploration.

In response to this comment, we have revised the wording in the main text and replaced “skills” with “areas of expertise” where applicable, as this more accurately represents the nature of the data collected. In our study, areas of expertise are reflected through the realms and sectors selected by stakeholders, as well as the projects and EU infrastructures in which they are actively engaged. These elements provided the basis for assessing network coverage and identifying areas where additional representation may be needed.

* You mention in the introduction "web application that allows users to monitor network changes and activity levels in real time". I would appreciate if you could comment on how realistic this proved to be, what changes are to be expected and which ones would be much harder to pick up (given that I presume users don't update their profile very frequently).

RESPONSE: We appreciate the reviewer’s comment and agree that, within the context of EuropaBON, we do not expect members to continue updating their profiles now that the project has officially concluded. However, since this network is intended to serve as a foundation for identifying partners for the envisioned EU Biodiversity Observation Coordination Centre (EBOCC), we anticipate that the network will remain active—or be reactivated—in the near future. The infrastructure developed through the EuropaBON member database and web application will be an important asset in supporting the establishment of the EBOCC. To help ensure the long-term sustainability of the network and its functionality, both the member portal and the dashboard were designed with long-term usability in mind and are planned to be maintained for at least five years beyond the project’s completion (i.e., until 2029).

* "Network members provided information essential to this analysis during the network registration process (<https://europabon.org/members/register/index>)." Note that the full set of questions the user is presented with are only shown upon actual registration, in the interest of this paper it would be useful to include/discuss these in more detail and have the full list included in the supplementary material.

RESPONSE: Thank you for the comment. We included the questions and possible answers in the supplementary material.

* The phrase "while filtering for verified users with non-empty data provider or data user fields" is unclear. Please elaborate.

RESPONSE: Thank you for this comment. We have removed this sentence as it does not appear to be particularly important in this section and added a rewritten version to the section “Position in the data flow”. Verified users refer to those who have completed email verification. Initially, the input fields for “data user” and “data provider” were optional, meaning entries in these fields were not required during the early stages of the project. As a result, in some cases no data was available for these fields in the initial registrations. They were only made mandatory at a later stage of the project.

* On the nodes API, the sentence "To establish edges, each value in the data user and data provider columns is iterated through, creating connections (from, to) via the nodes' IDs in the edges object." is not entirely clear. Please clarify.

RESPONSE: We thank the reviewer for raising this point and we apologise for not explaining this better. We have rewritten the text for the API to remove any ambiguities. The information on the API can now be found in the new sub-heading “Data processing”.

* S3 Figure: this figure seems rather unnecessary.

RESPONSE: Thanks, we agree this figure is rather unnecessary. We have removed this figure from the appendix.

* "Individual members' information is not disclosed in the dashboard and we grouped stakeholders by their affiliation (i.e., several individual members can belong to the same organisation and thus display as one and the same node)." Please specify how this was done, was this a simple 'distinct' operation?

RESPONSE: We have added a further sub-heading “Data processing”, in which we go into more detail about the processing of the data. We have also provided the complete SQL query for this table grouping as an attachment. The operation was a combination of “DISTINCT” and “Group by”.

* The section "Statistical analysis of network data" is very hard to read, also several components of the model lack explicit explanation in the text.

RESPONSE: The entire section “Statistical analysis of network data” has been rewritten. We hope it is much clearer now.

* "and OECD country outside within Europe" - Please check, probably this should be either read "outside" or "within"?

RESPONSE: Thank you for pointing this out. It should now read "and an OECD country outside Europe." We have made the correction.

* Table 1 - see earlier comment on nested/distributed organisations

RESPONSE: Please see our answer above.

*********************************************************************

**Reviewer #2:**

*********************************************************************

**General Comments**

This paper describes a network analysis identifying key EuropaBON stakeholders, their relationships, and participation patterns, and an associated interactive website that displays the data. The analysis is well executed and I have no suggested changes to that aspect. The website and analysis provide an effective model for other such networks to analyze and display participation patterns (even the larger GEO BON). Importantly, the authors provide full access to the code to reproduce the website and analysis, greatly facilitating such adoption.

My general criticism is that the Introduction and Discussion lack detail, including discussion of previous research, and analysis of other similar efforts to characterize conservation networks. That would allow the reader to appreciate how this analysis adds to our current understanding of the functioning of these networks. The Introduction is particularly sparse and contains some vague language about “data” and “communities”, perhaps under the assumption that the reader is already familiar with what types of data are collected in BONs and what types of players are involved.

I recommend adding detail throughout, with an eye towards making the story clear to readers with no familiarity with the concept.

The Introduction should ideally be expanded into at least 4 paragraphs.

Currently, it is lacking in citations and critical background information. The reader must wait until the Results and Discussion to understand why this dashboard is really important and needed. The central paragraphs should provide examples of why it would be useful to visualize this information and why this is currently an unmet need.

RESPONSE: We thank the reviewer for the constructive and encouraging feedback. We appreciate the suggestion to further highlight similar networks and communities, and fully agree that GEO BON serves as an excellent example of a global initiative advancing biodiversity monitoring. In response, we have expanded the introduction to include a broader context of other regional and national BONs, such as Asia BON, Americas BON, and SANBI, and added appropriate references to support this. We also emphasize that EuropaBON goes a step further by not only aligning with these global efforts but by introducing a dynamic and transparent network analysis tool. This allows us to actively assess and address gaps in geographic, thematic, and sectoral representation - an effort that distinguishes EuropaBON as a leader in promoting inclusivity and evidence-based improvements within the biodiversity monitoring community.

The Discussion should better highlight how learning about a network can improve it, and how subsequent improvements in the network translate to real conservation outcomes. I provide some suggestions to help assuage these concerns in the Detailed Comments below. Once these are addressed, I feel this paper will make a nice contribution.

**Detailed Comments

ABSTRACT**

The Abstract is good, but the description of what the dashboard actually displays is vague. Please add a few details in the final 6 sentences of the Abstract that give the reader some idea of specifically what types of data and information are displayed by the dashboard and how it might be used.

RESPONSE: We thank the reviewer for this comment and rewrote the abstract to include additional information on what the dashboard actually displays.

**INTRODUCTION**

1st paragraph, last sentence: “...accessibility of biodiversity data…”: What types of biodiversity data? Please give an example or two.

RESPONSE: We thank the reviewer for this helpful suggestion. To clarify what types of biodiversity data we are referring to, we have revised the final sentence of the first paragraph in the Introduction to include concrete examples. The sentence now reads: This joint effort seeks to build a unified and coherent system that improves both the reliability and accessibility of biodiversity data—such as species occurrences, habitat condition, and population trends—ultimately enabling more informed conservation strategies and evidence-based policy decisions.

2nd paragraph, 1st sentence: “...biodiversity communities…”: What is meant by this? Please be more specific.

RESPONSE: Thank you. We revised this paragraph and provided more information on similar biodiversity communities like GEO BON and other regional and national networks.

2nd par, 3rd sentence: “the impact of key contributors”: Again, I find this language to be a bit vague. What kinds of impacts are we talking about? Is this a matter of the amount of data that each country is contributing data to the BON? Please try to be more specific.

RESPONSE: Thank you. We clarified this in the text.

2nd paragraph of Introduction: This paragraph (ideally expanded to 2 or 3 paragraphs) should be where most of the background information is provided to the reader. However, it currently has no citations and is generally light on background information. Please expand this to provide the reader adequate background information so that they can fully understand the value of your dashboard contribution.

RESPONSE: Please see our answer above. We have expanded the introduction to include a broader context of other regional and national BONs, such as Asia BON, Americas BON, and SANBI, and added appropriate references to support this.

3rd paragraph of Introduction: “It offers high-level information in one view that can be used to identify occupational sectors, realms, or geographic regions with the most connections and pinpoint the central actors within the network.”:
You haven’t fully established why it is important to visualize the activities of the central actors, nor have you introduced why “occupational sectors” matter. Define what a “realm” is (e.g., how does it differ from a “sector”?).

RESPONSE: We have expanded the introduction by explicitly defining the three dimensions of analysis: occupational sectors (academia, NGOs, governmental organisations, private sector, citizen science, other), realms (terrestrial, freshwater, marine), and geographic regions (Northern Europe, Western Europe, Eastern Europe, Southern Europe, and non-European regions). The main objectives of this study were to map the EuropaBON stakeholder network across these dimensions and identify gaps in skills, thematic program areas, and geographic representation. Interactive graphs on the dashboard help to identify the most influential and active dimensions in shaping the future European biodiversity monitoring system.

“The main objective of this study was to 1) map the EuropaBON stakeholder network across sectors, realms and EU regions, identify 2) skills gaps, thematic-, and geographic gaps, 3) data providers and users 4) and key stakeholders, and 5) provide this as a fully responsive, interactive web application that allows users to monitor network changes and activity levels in real time.”:

There are logical problems with the flow of language in the above list of objectives including duplication and incorrect placement of verbs relative to list numbers. Rephrase to flow better, perhaps something like: “The main objectives of this study were to map the EuropaBON stakeholder network across sectors, realms and regions, and to identify gaps in skills, thematic program areas, and geography. Our aim was to create a fully responsive, interactive web application that allows users to monitor network changes and activity levels in real time.”

RESPONSE: Thanks, we have rewritten the sentence to: “The main objectives of this study were to map the EuropaBON stakeholder network across sectors, realms and regions, and to identify gaps in expertise, thematic program areas, and geography. Our aim was to create a fully responsive, interactive web application that allows users to monitor network changes and activity levels in real time.”

**METHODS**

Paragraph 1: The number of registered EuropaBON members etc. might fit better in the Introduction as background information.

RESPONSE: We agree with the reviewer and moved this sentence to the introduction.

Is “13.09.2024” a correct date format for this journal?

RESPONSE: Thank you for noticing. We have changed the date format into 13/09/2024 (Common in European and international English)

Paragraph 2: “Network members provided information essential to this analysis…” What kinds of information and in what form?

RESPONSE: We have added an appendix S2 which contains the complete questions of the registration form. We have added a reference to this appendix in the text.

Paragraph 3: “...through right joins in the database, combining information based on common identifiers…” This is unclear and perhaps provides too much detail without actually informing the reader of the end result. The same applies to this “...user details, event statistics, and ISO region information…”. It seems vague, limiting reproducibility, while at the same time providing little insight as to what actually was performed for a general reader. Also, spell out ISO.

RESPONSE: We have rewritten this section to clarify any misunderstandings.

“The API has four endpoints:”: Briefly explain here what an API endpoint is and what it does. Without that, the list of API endpoints is less meaningful. The links go to plain text pages which are backend data files that power the dashboard. But without a brief introduction to API endpoints, the reader is left confused about what the function of the links is.

RESPONSE: We addressed this comment in the text.

Bullet point 2: “storing them as nodes in JSON format”: Please explain a little what it means to be a node in JSON format. What do they mean in the context of the end user experience?

RESPONSE: We have rewritten the section to the API and added more information about the JSON-format. JSON stands for Javascript Object Notation, so it is basically a text format containing information as key-value pairs (eg., “name”: “iDiv”), where the key is “name” and the value is “iDiv”).

“Centrality” section: Perhaps add a little about what Centrality would mean in terms of EuropaBON members. The examples you provide about citations and page rank are illustrative but a more concrete example would be nice of what a highly central BON member would be like.

RESPONSE: We agree. We have added this information in the text about what centrality would mean in terms of EuropaBON members.

End of Page 7, beginning of page 8: Here is the first time you provide the subcategories in each category such as sector, realm, etc. These should be provided earlier so that it is clear what a realm, etc. is early on.

RESPONSE: We agree. We have added this information about the subcategories in the introduction and in the new sub-heading “Data processing”.

Beginning of page 8: “...several individual members can belong to the same organisation and thus display as one and the same node…”: I think this information should be made clear earlier on. Until now, it was not clear to me that nodes were organizations and that data from individual members in member portal were being aggregated to inform node characteristics.

RESPONSE: Thanks for the comment, we have moved this information to the new sub-heading “Data processing”.

Fig. 2 caption: All of these metrics are interesting sounding, but I can’t help but wonder what they will be used for and by who. The background and information regarding utility that is needed to make learning about these metrics interesting should be included in the expanded Introduction.

RESPONSE: The caption describes the components of the dashboard. We have added information on the value of analysing such metrics and the visualisation of the network on the dashboard in the expanded version of the introduction.

Page 9, penultimate paragraph of Methods: “...the number of EU directives associated with the stakeholder’s activities…”: I could be wrong, but wouldn’t the number of directives associated with the stakeholder’s activities be (to some extent) caused by the number of activities (the dependent variable) and not vice versa? I recommend carefully considering this possibility.

RESPONSE: We thank the reviewer for raising this important point regarding the potential for reverse causality between the number of EU directives associated with a stakeholder’s activities and the dependent variable (number of activities or degree centrality). The reviewer is correct to note that the number of EU directives could, to some extent, be influenced by the stakeholder’s level of engagement in activities, rather than solely acting as an explanatory factor. To address this concern, we carefully considered the nature of this variable during the model specification process. The number of EU directives was included as a proxy for the regulatory environment in which the stakeholder operates, reflecting the broader institutional context that may shape their engagement patterns. While reverse causality is a valid concern, we argue that the number of EU directives is more likely to represent a pre-existing condition that influences the stakeholder’s capacity or motivation to participate in activities, rather than being a direct outcome of their participation. However, we acknowledge that this relationship may not be strictly unidirectional. We also conducted a robustness check by re-estimating the model excluding the number of EU directives as a predictor. The results remained qualitatively consistent, suggesting that the inclusion of this variable does not unduly influence the overall findings. We have now explicitly noted this potential limitation in the revised manuscript.

Table 1: This table would be improved by explaining what centrality means and giving some indication of the range of values expected for this measure (e.g., it is impossible to know if the numbers displayed are high or low, relatively speaking).

RESPONSE: We have added information to the sub-heading “Centrality” to make it easier to interpret Table 1.

**RESULTS**

Second paragraph of Results: “...are clearly underrepresented in the network, with only 8% (n=44) of all stakeholders…”: This isn’t a supported statement without providing information about what % would be expected if they were proportionally represented. Please either provide this information or else revise the claim.

RESPONSE: We have revised this section in the text and provided additional information.

‘EuropaBON’s stakeholders’ section: “...research infrastructures, -networks, or -projects…”: The hyphens in this sentence are confusing. I suggest spelling out “...research infrastructures, research networks, or research projects…” if that is the intended meaning.

RESPONSE: We rephrased this sentence accordingly.

Fig. 4 caption: Please state what the colours represent.

RESPONSE: The colors in figure 4 have no special meaning. We have chosen the color scheme „viridis". It was designed to be colorblind-friendly and is popular in scientific visualisation tools. We have clarified this in the caption of this figure.

“Factors driving stakeholder…” section, first paragraph: You already state that you used negative binomial models in your Methods, so this is duplication. I would include this information only in the Methods. Much of this paragraph is duplicate information that already occurs (and belongs in) the Methods.

RESPONSE: We have revised this paragraph, and all duplications have been removed.

“Factors driving stakeholder…” section, second paragraph: “...these groups had on average by 2.4 and 1.3 more connections…”: I appreciate that you provide effect sizes here. Can you confirm that this means 2.4 and 1.3 more connections on average? Or is it 2.4 and 1.3 times more? Also, more than what, the average? The intercept group?

RESPONSE: We added a definition of marginal effects in the Method section that is:

“Marginal effects provide a quantitative interpretation of the model results by estimating the change in the expected value of the dependent variable associated with a one-unit increase in a specific explanatory variable, while holding all other variables constant [17]. This approach is particularly useful for understanding the practical significance of the explanatory variables, as it translates negative binomial regression coefficients into more interpretable units.”

And modified the results section, so we hope it’s clear now that in the case of Stakeholder type it means 2.4 and 1.3 more compared to the reference level which is the group of “Data users”. Here is the revised section:

“Stakeholders who acted as both data providers and data users, as well as those who solely acted as data providers, had significantly more connections compared to those who solely acted as data users. Specifically, these groups had, on average, 2.4 and 1.3 more connections than data users, respectively, with both effects being statistically significant at the 0.05 level.”

**DISCUSSION**

First paragraph: The sentence beginning “Our analysis…” is rather long and would benefit from editing to be more concise or by splitting it into two sentences.

RESPONSE: Thank you and we agree. We have addressed this accordingly.

Second paragraph: Can you better explain what the EBOCC is and what it would look like for it to focus on this network?

RESPONSE: Thank you and we agree. We have addressed this accordingly by expanding the text in the discussion.

Third paragraph: You set forth some measures to increase representation, which are good. But how would those translate into better outcomes? That piece is still lacking. Can you spell out why being well connected and inclusive matters?

RESPONSE: Thank you for this invaluable comment. We have added information about the applicability of this work to real-world problems in the respective section in the discussion..

Fourth paragraph: This paragraph starts out talking about realms, but finishes with a seemingly new topic (a list of key actors). Can you tie these together and provide a concluding sentence to this paragraph?

RESPONSE: We are very grateful for this comment and have addressed this in the text.

In general, there is a lack of cited literature in this Discussion and a corresponding lack of connection to previous research and scholarly discussion about conservation networks and planning.

RESPONSE: We have added references to other initiatives and research where applicable.

Last paragraph: You rightly point out that other similar collaborative efforts (including GEO BON) would benefit from a similar framework and analysis. I think you could brag a little here about how your solution provides a robust model (and even a complete code) to facilitate such widespread adoption. Well done.

RESPONSE: Thanks. We have added an additional paragraph to highlight the benefits a bit more.

**REFERENCES**

Again, I think the Introduction and Discussion could use more discussion of previous research and analysis of such conservation networks. That would allow the reader to appreciate how your analysis adds to our current understanding of these networks and how they operate.

RESPONSE: We have added references to other initiatives and research where applicable.

*********************************************************************

**Reviewer #3:**

*********************************************************************

Dear authors, dear editor,

The manuscript “The EuropaBON Stakeholder Dashboard: A dynamic web application to map Europe's biodiversity community” presents an innovative tool for mapping and analysing biodiversity stakeholder networks in Europe. By identifying key actors, highlighting data gaps, and enabling real-time updates, the dashboard contributes to advancing biodiversity monitoring and stakeholder engagement. While the manuscript aligns well with PLOS ONE's scope, it is not a traditional research article and deviates from the journal's preferred structure. Additionally, the language may be overly technical for a general audience. Although the contribution is valuable, it lacks full originality due to the existence of similar tools in other domains (e.g., biodiversity and environmental networks).

The manuscript contributes to biodiversity monitoring and stakeholder engagement.
The interactive dashboard and open-access code enhance transparency and usability, but the overall work and presentation lacks balance in terms of representation and clarity on how to overcome these issues, e.g. targeted outreach strategies to address geographic or realm underrepresentation. An outlook on the further maintenance is also missing (“at least five years after the project’s life time is a bit vague)

**General comments:**

A key concern is the lack of clarity regarding how stakeholders became members of the network. Did the authors actively approach key biodiversity actors, or was recruitment primarily passive (e.g., through social media outreach)? The absence of notable stakeholders, such as certain organisations in Switzerland or prominent institutions like the Natural History Museum in London, raises questions about the representativeness of the database. Additionally, incomplete entries (e.g., NHM Vienna) further suggest a lack of systematic inclusion.

RESPONSE: Thanks. We have added some more information in the section “Input data”.

Similar concerns arise regarding the measurement of activity levels. How were stakeholders invited to participate in events, and how did the authors account for potential barriers, such as funding constraints or scheduling conflicts? A lack of participation in specific EuropaBON events does not necessarily indicate lower overall engagement in biodiversity-related activities (only because one specific date would not suit them, does not make them less active in the field…).

RESPONSE: Thanks. We have added some more information in the sub-heading “Activity levels”.

Additionally, the visual use of red to indicate participation below a threshold (e.g., fewer than 8 events) could be misleading, as red is often associated with negative connotations like "alarm" or "failure."

RESPONSE: Thank you for spotting this. We agree that the color red is misleading. We have changed the color into yellow, the colors (red = none, yellow = 1-8 events , green > 8 events) for the activity levels match now the color scheme in the table below.

Another major concerns is how the member data are updated; from experience it would need regular reminders to ask members to update their data (including someone sending these reminders and chasing these data); generally I think the requested data do not change too often (except maybe projects organisations are involved in) and they are updated maybe only once a year (maximum), so that it is not really relevant to have this real time component; that of course takes a lot of time when using and filtering the dashboard.

RESPONSE: Thank you for your comment. Implementing a real-time component is standard practice in modern web applications. Traditionally, data was pulled directly from a database, whereas today, APIs are commonly used to retrieve and display the most up-to-date information dynamically. The goal of this project was to develop a web application based on the existing raw data from the EuropaBON members portal, transforming it into a structured, interactive, and user-friendly dashboard. Whether users actively update their data is secondary; the primary focus is on processing the raw data and making it FAIR (Findable, Accessible, Interoperable, and Reusable) through a web interface and API. This approach distinguishes itself from traditional network analyses, which have typically been conducted in a static, internal manner, without online accessibility or interactive features. By contrast, our method ensures greater transparency, usability, and engagement with the data.

Some of the terminology is not consistent. In the introduction authors talk about “the system”, “the network” and it is not clear what is meant and when it relates to the stakeholder network and/or the biodiversity observation system. This should be checked throughout the manuscript.

RESPONSE: The EuropaBON project, the biodiversity monitoring system, and the stakeholder network are distinct entities. We have clarified this distinction in the introduction and used the terms ‘project’, ‘system’ or ‘network’, depending whether we refer to the EuropaBON project and its associated aims and activities, the integrated biodiversity monitoring system being developed by the project for Europe, or the growing network of stakeholders and members engaged throughout the project.

Line 64 says “in developing a EuropaBON”: here is a mixture between the European BON (the regional BON of GEO) and EuropaBON (the project); if the same name is used for both, then this should be clarified somewhere; the entire sentence does not make too much sense and needs to be rephrased. What is the “collaboratively designed system”?

RESPONSE: Thank you for the comment. Yes, both the monitoring system, as well as the project are referred to as EuropaBON. We have clarified this in the first paragraph of the introduction.

In line 60 it is stated that network members were involved in developing the new European biodiversity monitoring system: if this is already in place, then this would need a clear reference! See also 169 onwards.

RESPONSE: No, this system is not yet in place. Recommendations for the design of this system can be found in Kissling, W. D., Breeze, T. D., Liquete, C., Solheim, A. L., McCallum, I., Maes, J., et al. (2024). Towards a modern and efficient European biodiversity observation network fit for multiple policies. EcoEvoRxiv. https://doi.org/10.32942/X2WW2H. We have clarified this in the text.

**Methods:**

Including a table summarising the information queried during registration would significantly enhance clarity. Additionally, a brief overview of the database structure, including key tables and their relationships, would help readers better understand how data were organized and processed. For instance, the statement “we retrieved data from multiple tables (users, profile, events, country) through right joins in the database, combining information based on common identifiers” is overly technical and could confuse non-specialist readers. Simplifying or illustrating this process would improve accessibility.

RESPONSE: Thank you for the comment. We have included a full list of questions from the registration form in the appendix. We have also rewritten the text for the API for better understanding. The API is more or less the mirror of the database tables in a structured way using key-value pairs. Therefore we believe that the API is sufficient to understand how the data is organised.

The API is described in detail (at least endpoint 2), which might be too specific for the reader and the journal after all. I think the description of the API is not really relevant for the paper and could be moved to the supplementary material. “nodes” and “edges” should be explained to the non-technical reader and the paragraph should be checked for readability and understandability.

RESPONSE: See comment above. The API can be seen as the public version of the database, so we think it's important to show how the data we display in the dashboard is organised. We have rewritten the section on the API, we hope this will remove any ambiguity.

For people not familiar with network analysis the terms “centrality” and “page rank” need to be introduced before discussing them.

RESPONSE: The term “centrality” and “page rank” are both described in detail in the sub-heading “Node properties”, therefore we see no reason to go into further detail here.

Regarding the position in the dataflow it would be helpful if the data flow could be depicted in a figure.

RESPONSE: The positions in the data flow (data user, data provider, both) are shown in the network graph on the website (show network by sector/realm/EU region) and in Figure S8.

We have also made it clear again in the text that the edges (i.e., the connections) in the network graph are based on the position in the data flow.

Regarding the functionality of the dashboard it would be cool if one could search for an organisation in the last table, then click on it and see it in the first map; in that map it would be nice to be able to click on the connections as well and get an information where it links to (this is especially useful if you are zoomed in). Also the number of connections of an organisation could be shown in the mouse-over box.

RESPONSE: Thank you for this great suggestion. Unfortunately, as the project has ended, there are no remaining resources available to implement these features.

**Results:**

Some of the results are clearly connected to the question above of how people were asked to join; the underrepresentation of certain realms could be a result of not having actively asked freshwater or marine people. In that aspect, only recording “cross-realm” (without knowing which realms are crossed) also biases the evaluation.

RESPONSE: We agree that member recruitment may have been biased toward certain realms or European regions. The composition of the consortium naturally plays a significant role in shaping the stakeholder community. In designing the consortium, we made efforts to ensure balance by including, for example, partners from Eastern Europe as well as freshwater and marine experts. We acknowledge this issue in the discussion section of the manuscript, where we note: “The current low levels of Eastern European participation in the network are an important point to address. Additional efforts could be made here in terms of outreach, including the organisation of meetings, conferences, and workshops in Eastern Europe.”

The point regarding cross-realm representation is particularly important, and we will take it into account when extending the analysis, for example in the context of developing the anticipated EBOCC.

The headline “Factors driving stakeholder centrality and activity” does not correspond with what is really in this paragraph and I would recommend to rename this using a more common word than “centrality”.

RESPONSE: We replaced the headline “Factors driving stakeholder centrality and activity” by

“Factors explaining stakeholder impact and engagement”.

**Discussion:**

The discussion would benefit from a more critical analysis and a broader integration of references beyond EuropaBON-related publications. For example, statements such as “the network would greatly benefit from enhanced interaction with the freshwater and marine communities to exchange on many points of common interest” are vague and lack concrete suggestions for achieving these goals. Similarly, while the importance of ensuring the network's longevity is emphasised, the manuscript does not provide specific strategies or mechanisms for sustaining the network beyond the project’s timeline.

All of my above-mentioned concerns need to be thoroughly addressed in the discussion including recruitment, activity level, maintenance etc.

RESPONSE: We thank the reviewer for these comments. We have revised the discussion substantially and added multiple references to address these comments accordingly.

Additionally, a short discussion on “Stakeholders acting as both data provider and data user, as well as those solely acting as data provider, had significantly more connections compared to those solely acting as data users.“ would be nice as I think this is a valuable result. Also should it be discussed why it is not surprising that GBIF has the most connections.

RESPONSE: Yes, we agree with this comment. We have added a paragraph in the discussion to highlight this result and emphasize that GBIF serves as a key data provider, facilitator, and platform for effective biodiversity data management and integration.

Further, the authors could strengthen the case for originality by explicitly comparing their tool with existing platforms.

RESPONSE: Thank you very much for this positive comment. We agree and have addressed this comment both in the introduction and discussion.

Language wise the paper should be reviewed by a native speaker. Also the use of tenses needs to be re-checked (mixture of past and present tense in the methods for example)
In terms of used citations I would appreciate having a more balanced amount of non EuropaBON references.

RESPONSE: We thank the reviewer for this comment. The entire manuscript has been reviewed again for clarity and readability by a native English speaker (one of our co-authors).

**Specific comments:**

Line 29: rephrase as “one of the most comprehensive networks of biodiversity stakeholders to date was recently developed…”

RESPONSE: We addressed this comment in the text.

47: add that there is also a separation in terms of the different realms/biomes

RESPONSE: We addressed this comment in the text.

63: the EBV will not be monitored by the system but rather by the stakeholders… maybe “within the system”

RESPONSE: Thanks for the comment. We rephrased the sentence accordingly.

64: “in developing a EuropaBON”: here is a mixture between the European BON (the regional BON) and EuropaBON (the project); if the same name is used for both, then this should be clarified somewhere

RESPONSE: See response to previous comment. We addressed this in the text.

68-73: these are results and should be skipped here

RESPONSE: We have revised this entire paragraph to make it short and concise and removed results on the dashboard.

69: what is meant by “displays of Europe's biodiversity community”?

RESPONSE: Thanks for the comment. We rephrased the sentence accordingly.

73 onwards: rethink the punctuation and rephrase as “The main objectives of this study were 1) to map the EuropaBON stakeholder network across sectors, realms and EU regions, 2) to identify skills gaps, thematic and geographic gaps, 3) to identify data providers and users, 4) key stakeholders, as well as 5) to provide this as a fully responsive, interactive web application that allows users to monitor network changes and activity levels in real time.” Or something along these lines

RESPONSE: We addressed this comment in the text.

88: add examples which information was queried

RESPONSE: We have added an attachment S2 which contains the complete questions of the registration form.

93: what are verified users?

RESPONSE: We addressed this comment in the text.

95: skip “server’s”

RESPONSE: We addressed this comment in the text.

97: I would think the API is not part of the input data and should have a sub-heading on its own

RESPONSE: Yes we agree, we have summarized the text for the API as a new sub-heading “Data processing”.

101: which table?

RESPONSE: We have rewritten the text on the API. The table referred to the final nodes table, which summarizes all institutions after grouping.

148: please make clear if the web application is the base for the dashboard or if the web application IS the dashboard

RESPONSE: We addressed this comment in the text.

154: say that MariaDB actually is a database

RESPONSE: We addressed this comment in the text.

157: add “(Germany)” next to iDiv

RESPONSE: We addressed this comment in the text.

168: harmonise the use of capital letters for northern, western etc. (it is used with capital letters later on, eg line 198); the hyphen after northern-, western-, eastern- needs to be skipped

RESPONSE: We addressed this comment in the text.

186: what does this mean “it can often be modelled”?

RESPONSE: The entire section has now been rewritten, and we hope it is clear. By stating "*it can often be modelled,*" we meant that the negative binomial model is a statistical tool frequently used to analyse the type of data under consideration.

193: harmonise “PageRnk” vs “page rank”

RESPONSE: We addressed this comment in the text.

198 and everywhere else: harmonise the use of a/no comma after “i.e.” and after “e.g.”

RESPONSE: We addressed this comment in the text.

200: harmonise the use of capital letters for the organisations as well throughout the manuscript

RESPONSE: We addressed this comment in the text.

202: EU Directives: where does this information come from?

RESPONSE: The information on the EU directives was requested during registration. We have added an attachment S2, which contains the complete questions of the registration form.

Table 1: spell out BOLD as well; isn’t it “BOLDSYSTEMS” now?

RESPONSE: Thanks for spotting this. We have changed the name to “Barcode of Life Data System (BOLD)” which is the official name according to wikipedia.

222: remove the space after the “/” and harmonise this throughout

RESPONSE: We addressed this comment in the text.

224: skip “currently located in this region”

RESPONSE: We addressed this comment in the text.

225: remove the hyphen

RESPONSE: We addressed this comment in the text.

236: remove the hyphens

RESPONSE: We addressed this comment in the text.

249: remove the hyphen

RESPONSE: We addressed this comment in the text.

Figure 4: explain the colours

RESPONSE: The colors in figure 4 have no special meaning. We have chosen the color scheme „viridis". It was designed to be colorblind-friendly and is popular in scientific visualisation tools.

257: could you give an example for the overdispersion?

RESPONSE: We have modified the entire section. We also added the explanation of what overdispersion is.

“*This model is particularly well-suited for addressing overdispersion, a common issue in count data where the variance exceeds the mean, leading to underestimated standard errors and inflated significance levels in Poisson models. The negative binomial model accounts for this by introducing an additional parameter to model the excess variance, providing a more robust and flexible alternative to the Poisson model [15].*”

259: skip “respectively“

RESPONSE: The entire section has now been rewritten, and the word respectively has been removed.

258-261: this is methods and should be moved there

RESPONSE: The entire section has now been rewritten, and the parts that were methodological in nature have either been moved or removed to avoid duplication.

271: rethink the use of “better” rather than “more” as better implies that the quality is higher while it is only the count of connections!

RESPONSE: We have replaced "better" with "more," as suggested. Thank you.

299: to which other networks did you compare this? This should be added.

RESPONSE: We have clarified and addressed this in the discussion.

312: add a second intergovernmental organisation; GBIF to…

RESPONSE: We addressed this comment in the text.

314: remove the hyphen

RESPONSE: We addressed this comment in the text.

320: remove the hyphen

RESPONSE: We addressed this comment in the text.

322: I suppose it should be JRC (Joint Research Centre)

RESPONSE: We addressed this comment in the text.

334 onwards: please rephrase this sentence; there are five (!) “and” in there…

RESPONSE: Thank you for this comment. We have revised this sentence.

338: add other initiatives that could be relevant

RESPONSE: Thank you very much. We have addressed this in the discussion.

340: replace “these initiatives“ by “this initiative”

RESPONSE: We addressed this comment in the text.

344: “synergies” instead of “synergy”

RESPONSE: We addressed this comment in the text.

345: I do not understand “recruit non-members” as they will be members as soon as they are recruited, no?

RESPONSE: Thank you for this comment. We have revised this sentence. Nodes in the network that are marked as “non-members” are institutions that are not members of EuropaBON but are listed as data providers and/or data users of members.

346: consider using an alternative term to “networks” here as generally “network” is mostly used as EuropaBON network in this manuscript (as at the end of this sentence)

RESPONSE: We addressed this comment in the text.
