# Supplementary material for: The EuropaBON Stakeholder Dashboard: A dynamic web application to map Europe’s biodiversity community
Source: PLoS One. 2025 Aug 13;20(8):e0329390. doi: 10.1371/journal.pone.0329390 (PMC12349692; doi:10.1371/journal.pone.0329390)
Supplement: Attachment — Submitted filename: Response_to_Reviewers_auresp_2.docx [file pone.0329390.s013.docx]

Response to Reviewers

*********************************************************************

**Reviewer #3:**

*********************************************************************

Still, I do have some concerns regarding the publication of the paper.

A lot of time and effort was invested in building up the dashboard and in writing this manuscript. But the sustainability of the dashboard is still very vague and needs to be improved.

As I read it now, the dashboard should be further used for the EBOCC. The paragraph on EBOCC introduces a potentially important initiative but remains vague. It is unclear whether EBOCC is a formal commitment or a proposal under review, and how it relates to the future of the EuropaBON dashboard and stakeholder database. If the intention is to transfer or integrate these components into EBOCC, this should be stated more clearly. Otherwise, the long-term sustainability and purpose of the dashboard remain uncertain.

RESPONSE: Thank you for this valuable comment. We agree that greater clarity regarding the status of the European Biodiversity Observation Coordination Centre (EBOCC) and its relationship to the EuropaBON dashboard is important. We have therefore revised the paragraph to state that the EBOCC has recently been launched as a call for tenders by the European Commission's Directorate-General for Environment. The EBOCC is not merely a proposal, but an initiative now entering its implementation phase. As outlined in the revised text, the EBOCC aims to pilot and test a coordinated biodiversity observation centre, building directly on the framework and tools proposed by EuropaBON. This includes the potential further development and integration of key components such as the EuropaBON dashboard and stakeholder database. These elements are thus envisioned as foundational assets for the EBOCC, contributing to its functionality and long-term sustainability.

Also, the manuscript could benefit from explicitly stating whether the EuropaBON stakeholder network is still open to new members and how interested individuals or institutions can join. Since the dashboard is promoted as a real-time tool, clarity on ongoing recruitment and integration of new members is essential for demonstrating its long-term relevance and inclusivity.

RESPONSE: Thank you for your comment as well. We have added a section to the discussion - “Opportunities and limitations of the dashboard” - to better clarify its long-term relevance and inclusivity.

The claim that the dashboard is reusable and could be adopted by other networks is valuable but currently vague. The manuscript should specify which components are reusable (e.g., code, database structure, API), what skills are required to adapt them, and whether any documentation or support is available. If establishing “connections with our network” refers to API-level interoperability or shared infrastructure, this should be explicitly described.

RESPONSE: We have revised the manuscript to clarify which components are reusable. Specifically, we now state that the source code is openly available via the project’s GitHub repository and that the public API allows programmatic access and interoperability. We have also specified that "connections with our network" refers to API-level interoperability, enabling external systems to link directly to our network infrastructure.

Additionally, we noted that further support and guidance are available upon request, with continued maintenance of the dashboard and portal infrastructure planned until at least 2029. These clarifications are now included in the revised text to provide a clearer understanding of how the dashboard can be reused and integrated by other networks.

In terms of language and length, I think some sentences are too lengthy and written too detailed (I even suggested some removements). The methods chapter is far too long. This is a scientific article and not a how-to-manual on building up an API or dashboard. The API endpoints and technical architecture are overly technical for a general audience. SQL queries, API responses, name-value pairs or examples for the attributes can be skipped or moved to Supplementary Material.

RESPONSE: Thank you, we have revised the methods chapter and moved the information about the API endpoints to the supporting information. We have deleted individual sentences that seemed too technical as well, the methods chapter only contains basic, technically important information now.

Some information is just not relevant (“It was developed by the original…”) and should be skipped.

RESPONSE: We skipped the sentence “It was developed by the original…” accordingly.

The discussion is still very superficial in terms of practical implications. It still touches too lightly on critical points like dashboard maintenance, integration with other networks, and tangible next steps (see all my entry points). It repeats results at times (e.g., GBIF's centrality) instead of building on them and the tone is largely promotional.

For better readability, the discussion should be dived into subchapters, e.g.

• Inclusiveness and representation in the Network

• Role of key institutions and central actors

• Implications for biodiversity monitoring and policy

• Opportunities and limitations of the dashboard

• Future directions and recommendations

RESPONSE: We appreciate this thorough and constructive feedback. In response, we have substantially revised and restructured the discussion section to more clearly address the practical implications of our findings. Specifically, we have reorganized the discussion under the subheadings suggested by you, which has improved both the readability and depth of the discussion.

Still some tense issues are remaining, mixing up use of different tenses in the introduction and methods (e.g. “EuropaBON advances this work by offering a dynamic, continuously updated real-time tool for exploring network structure and engagement, drawing on the FAIR principles” should be in past tense as the project is over.).

RESPONSE: Thanks, we have corrected the grammar of the paper once again, also the above sentence has been corrected.

Also the use of capital letters for attributes or also for the directives needs to be harmonized.

RESPONSE: We have carefully checked the manuscript and corrected some inconsistencies.

However, we would like to clarify one point regarding the formatting of the API: The keys (i.e. attributes) are written in lowercase for technical reasons, which is standard JSON formatting practice. In contrast, the values associated with these keys reflect the predefined response options provided during the stakeholder registration process.
These values retain their original formatting, which include capitalisation for EU directives (e.g. “Climate Adaptation Strategy”) or for the occupational sector (e.g. “Academia”) as defined in the registration form.
As the API is the basis for the data in the dashboard, it is therefore possible that there are still some inconsistencies in the dashboard visualisation compared to the manuscript. However, these inconsistencies can be corrected in the future as the tool is further developed.

**Specific comments**

**Introduction**

2nd paragraph: “… promotes the use of Essential Biodiversity Variables (EBVs)…”: explain what the EBVs can be used for (still not clear to everyone…)

RESPONSE: We have added additional information about the EBVs to explain them better.

“… have been involved in every step of the development of the new European biodiversity monitoring system…”: this still sounds like the system is already in place; maybe start with explaining what and how far it is (“Although not yet implemented…”) and then add the info how EuropaBON members where involved; please also add the general aim of the new system.

RESPONSE: We addressed this by rewording these two sentences in the introduction.

“their participation in project activities”: please change to “EuropaBON project activities”

RESPONSE: We addressed this comment in the text.

“Northern Europe, Western Europe, Eastern Europe, Southern Europe, and non-European regions”: this reads a bit weird as there is no Central Europe, but I guess I should have commented on this earlier…

RESPONSE: We acknowledge that the categorisation may give the impression that “Central Europe” is not considered as a region in its own right. However, for the purposes of this study, we follow the United Nations geoscheme, which classifies countries commonly associated with Central Europe under either Western Europe or Eastern Europe. This approach ensures consistency with international regional classifications and allows for clearer comparative analysis across the defined EU regions.
However, we have added additional information in the text.

**Methods**

Input data, 1st paragraph: the very first sentence about the data cleaning is rather lengthy and also the example of only one input field is confusing; you could simply state something like

“To ensure consistent institutional representation on the dashboard, we grouped individual member entries by their affiliated institution. When multiple members from the same institution provided different roles in the data flow (e.g., one as a data user, another as a provider), we combined these responses to classify the institution accordingly (e.g., as both user and provider). …”

or something along these lines

RESPONSE: Thanks. We rephrased the beginning of this section accordingly.

Data Processing: Network graph:

“Each node is assigned an ID and corresponding attributes (e.g. “id”: “1”, 'label': “German Center for Integrative Biodiversity Research (iDiv)”, "scope": "Global", "group": "datauser", etc.).”: I would skip the example and just list the attributes; please check the use of different types of inverted commas as well as the capital letters in the attributes (see also Terrestrial, Freshwater below).

RESPONSE: We addressed this comment in the text.

“The centrality values for each individual node are calculated after the creation of the API and are therefore not included in the endpoint. The appendix S5 file shows an example of the attributes for ID 1.” Skip the first part and move the reference to S5 above to the ID section.

RESPONSE: We addressed this comment in the text.

Start a new paragraph at “The API endpoint also contains information about the connections (edges) between the individual nodes.” The sentence is hard to read, maybe rephrase as “This API endpoint also contains information about the connections between the individual nodes, represented by the edges in the network graph.”

RESPONSE: We rephrased this sentence accordingly.

Node properties, Centrality, 2nd paragraph: skip the sentence “It can therefore be said that a highly…”

RESPONSE: We addressed this comment in the text.

**Statistical model of network data:**

the first sentence says something about the aim of the section (i.e. the analyses); but the aim of the section is to explain the methods! Please skip or change.

RESPONSE: We have revised the beginning of the section. The revised sentence now reads:
 “This section describes the statistical methods used to examine the factors associated with degree centrality and the number of activities in which EuropaBON members participated. Since both outcomes are count variables (i.e., non-negative integers), we considered a range of count regression models [27,28].”

“To identify the most suitable approach, we evaluated multiple statistical models …” skip that sentence.

RESPONSE: The section was rewritten and the sentence was removed.

“including non-OECD country outside of Europe, or OECD country within Europe”: I am not clear why this OECD/non-OECD approach was chosen; this should be explained, e.g. “We distinguished between OECD and non-OECD countries outside Europe to account for potential differences in institutional capacity and access to biodiversity infrastructure, which may affect participation and connectedness within the network.”

RESPONSE: Thank you for this comment and suggested clarification. We have revised the manuscript accordingly.

“The number of EU directives was included as a proxy for the regulatory…”: please indicate the exact purpose of why and how this question was included in the registration process (“Which EU Directives are relevant for your work?”) otherwise this sentence here is a bit unclear as the Directives are not related to the institutions.

RESPONSE: We have revised the text for clarity. It now reads:

“The directives variable was based on responses to the question, “Which of these EU Directives is of most interest to your work?”, which was included in the registration process to better understand the policy contexts informing participants’ expertise and focus areas.

**Results**

EuropaBON’s network, 2nd paragraph: skip “(in order of descending abundance)”.

The sentence “The low number of citizen scientists may…” is confusing, redundant, and speculative and should be changed; suggestion: “Only seven members identified their primary affiliation as citizen science. However, it is possible that more individuals involved in citizen science chose to register under another institutional category, such as academia or NGOs.”

RESPONSE: We rephrased this sentence accordingly.

Skip the end of the sentence “suggesting that the region is underrepresented relative to its political and geographic footprint in Europe.”

RESPONSE: We addressed this comment in the text.

EuropaBON’s stakeholders, 1st paragraph: add “highest centrality” and “highest page rank”

RESPONSE: We addressed this comment in the text.

Figure 3 caption: skip “The visualisation was generated with Cytoscape v3.10.2 [30] using the Compound Spring Embedder (CoSE) layout algorithm [31].” This is Methods.

RESPONSE: We moved this sentence to the sub-heading “Web application architecture” in the methods.

Figure 4 caption: skip “We selected the "viridis" color scheme…” as this is not relevant. Keep the last sentence though.

RESPONSE: We addressed this comment in the text.

Factors influencing stakeholder impact and engagement: change the chapter title to “Stakeholder impact and engagement”

RESPONSE: We addressed this comment in the text.

1st paragraph: skip the last sentence “We begin by….”; this is needless.

RESPONSE: We skipped this sentence accordingly.

**Discussion**

Please see all my comments above.

RESPONSE: We have rewritten the entire discussion to address all your comments above.

2nd paragraph: The sentence “Institutes classified as "cross-realm" - those involved in more than one realm - make up the majority of network nodes. This is primarily because, during data processing, information from individual members affiliated with the same institution was consolidated. When members listed different realms for the same institution, these were grouped under the "cross-realm" category. Of course, it is also possible that many institutions today genuinely operate across multiple realms, reflecting the increasingly interdisciplinary nature of research and practice [32].”

is confusing and could be improved, e.g. through “The large proportion of ‘cross-realm’ institutions results in part from data processing: when different members of the same institution indicated different realms, their entries were consolidated. However, this may also reflect a genuine trend toward interdisciplinarity, as many institutions increasingly engage across multiple ecosystem types [32].”

RESPONSE: Thanks, we rephrased this sentence accordingly.
